# A nine-atom rhodium–aluminum oxide cluster oxidizes five carbon monoxide molecules

**DOI:** 10.1038/ncomms11404

**Published:** 2016-04-20

**Authors:** Xiao-Na Li, Hua-Min Zhang, Zhen Yuan, Sheng-Gui He

**Affiliations:** 1 Beijing National Laboratory for Molecular Sciences, State Key Laboratory for Structural Chemistry of Unstable and Stable Species, Institute of Chemistry, Chinese Academy of Sciences, Zhongguancun North First Street 2, Beijing 100190, China; 2 University of Chinese Academy of Sciences, Beijing 100049, China

## Abstract

Noble metals can promote the direct participation of lattice oxygen of very stable oxide materials such as aluminum oxide, to oxidize reactant molecules, while the fundamental mechanism of noble metal catalysis is elusive. Here we report that a single atom of rhodium, a powerful noble metal catalyst, can promote the transfer of five oxygen atoms to oxidize carbon monoxide from a nine-atom rhodium–aluminum oxide cluster. This is a sharp improvement in the field of cluster science where the transfer of at most two oxygen atoms from a doped cluster is more commonly observed. Rhodium functions not only as the preferred trapping site to anchor and oxidize carbon monoxide by the oxygen atoms in direct connection with rhodium but also the primarily oxidative centre to accumulate the large amounts of electrons and the polarity of rhodium is ultimately transformed from positive to negative.

## Abstract (web summary)

Noble metals can promote the stable lattice oxygen of metal oxides to take part in oxidation reactions. Here, the authors report the preparation and reactivity of rhodium-aluminum oxide cluster ions, in which the rhodium ion promotes the transfer of five oxygen atoms from a nine-atom parent cluster to oxidize carbon monoxide.

## Graphical abstract

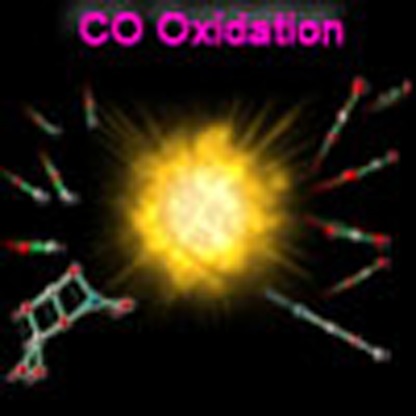

Oxide-supported rhodium (Rh) exhibits extraordinary catalytic activity in a large number of reactions^1^,^2^,^3^,^4^,^5^,^6^,^7^,^8^,^9^ such as the oxidation of carbon monoxide (CO)^1^,^2^,^3^,^7^, carbon dioxide methanation^6^, partial oxidation of methane to syngas^4^,^5^,^8^,^9^ and so on. It has been reported that trace amounts of Rh can promote direct participation of lattice oxygen of chemically very inert supports such as aluminum oxide (Al_2_O_3_), to oxidize reactant molecules^4^,^5^,^9^, while the fundamental mechanism is elusive. Exploring the function of Rh in invoking the lattice oxygen of oxide support is of great importance to understand heterogeneous catalysis but remains a big challenge because of the structure complexity of bulk material.

Atomic clusters are considered as the intermediate matter to bridge atoms and their bulk counterpart^10^, and can be ideal models for active sites of condensed-phase system. Cluster reactions^11^,^12^,^13^,^14^,^15^,^16^,^17^ can be studied under isolated conditions to provide the mechanistic insights of elementary steps in the related condensed-phase systems. Important findings such as spin conservation^13^ and the complementary active sites^15^ have been revealed by studying the reactions of aluminum clusters with molecular O_2_ and water, respectively. The oxygen atom transfer (OAT) from metal oxide clusters to small molecules is one type of extensively studied reactions^12^,^18^,^19^. Noble metal-doped heteronuclear oxide clusters^20^ are being actively studied to understand the mechanistic nature of supported catalysts in the OAT reactions such as CO oxidation, an important model reaction in heterogeneous processes^21^ and its wide applications in air purification. Au and Pt atoms have been emphasized to be crucial, to promote significantly the efficiency of OAT in CO oxidation^22^,^23^,^24^,^25^,^26^. However, each of the reported Au or Pt-doped clusters such as AuAl_3_O_5_^+^ and PtAl_3_O_7_^−^ can transfer at most two oxygen atoms to oxidize CO (refs 22, 23, 24, 25, 26). Here we report that a single Rh atom can unexpectedly promote the transfer of five oxygen atoms to oxidize CO from a nine-atom cluster RhAl_2_O_6_^+^, which produces the oxygen very deficient species RhAl_2_O^+^. In contrast, reported homonuclear aluminum oxide clusters (Al_*x*_O_*y*_^±^)^26^,^27^ can deliver only one oxygen atom to CO and these reactive clusters such as Al_2_O_3_^+^ and Al_4_O_7_^−^ are all oxygen-rich species. Identification of multiple OAT from a single Rh-atom-doped cluster to reactant molecules is an important step to understand the participation of lattice oxygen promoted by noble metals. This gas-phase study that a nine-atom rhodium–aluminum oxide cluster oxidizes five CO molecules is a sharp improvement in the field of cluster science and provides a strictly molecular level understanding of the fundamental mechanism of noble metal catalysis in the related condensed phase.

## Results

### Reactivity of rhodium–aluminum oxide clusters with CO

**Figure 1:**
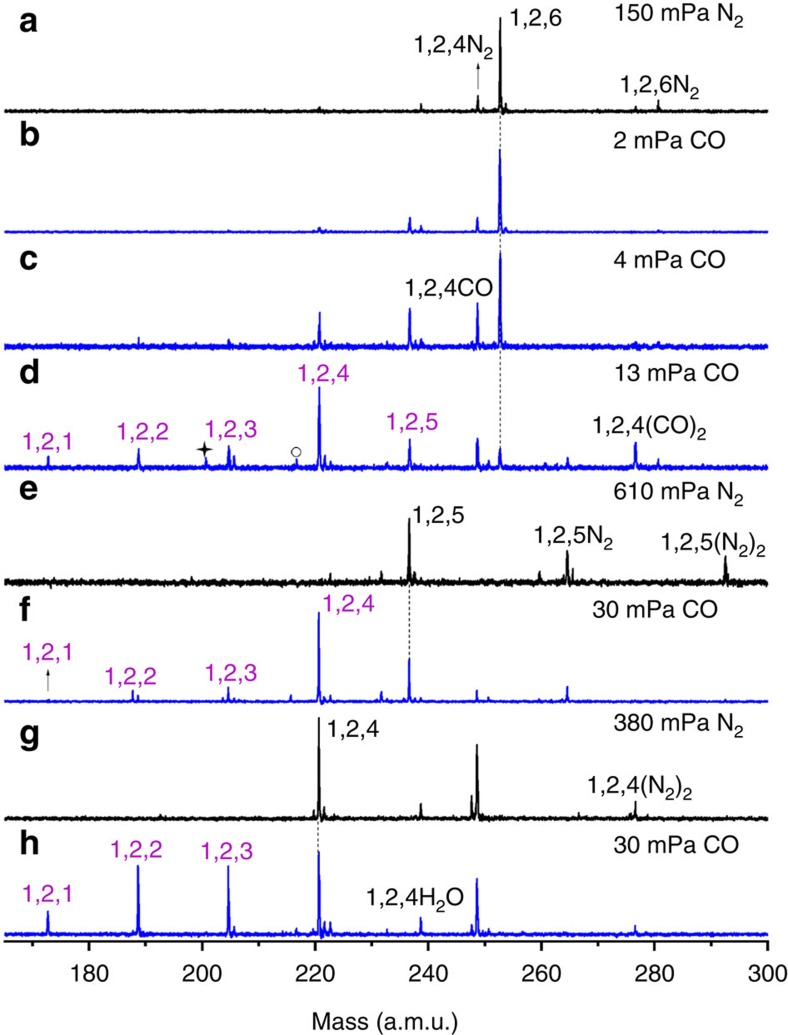
Reactivity of RhAl_2_O_4–6_^+^ clusters with CO. Time-of-flight mass spectra for reactions of mass selected RhAl_2_O_4–6_^+^ with N_2_ (**a**,**e**,**g**) and CO (**b**–**d**,**f**,**h**) are shown. Peaks marked with asterisk and hollow circle in **d** are CO adsorption products of RhAl_2_OCO^+^ and RhAl_2_O_2_CO^+^, respectively. Rh_*x*_Al_*y*_O_*z*_^+^ and Rh_*x*_Al_*y*_O_*z*_*X*^+^ (*X*=N_2_, CO and H_2_O) species are labeled as *x,y,z* and *x,y,zX*, respectively. Signal 1,2,4H_2_O in **h** is due to the residual water in the gas handling system. The time periods for reactions RhAl_2_O_6_^+^ + CO, RhAl_2_O_5_^+^ + CO and RhAl_2_O_4_^+^ + CO were about 1.1, 0.7 and 0.6 ms, respectively. The reactant gas pressures are shown in mPa.

**Figure 2:**
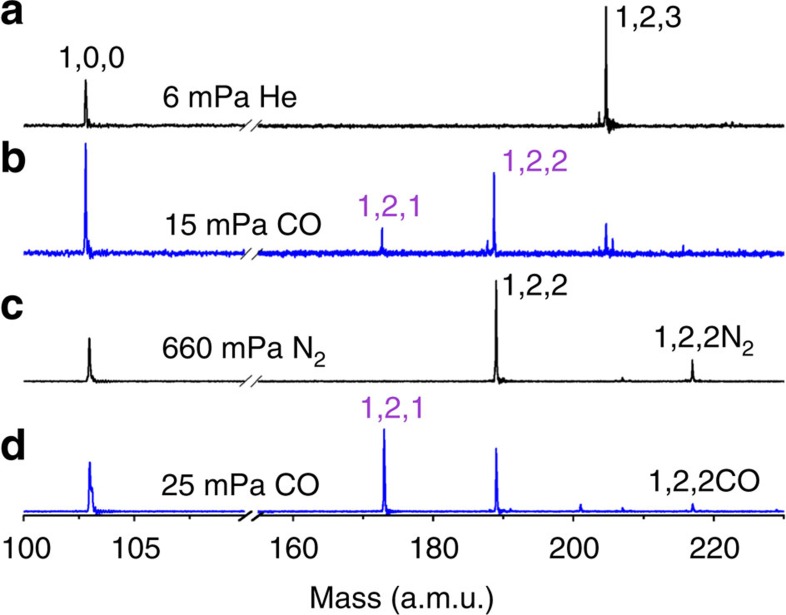
Reactivity of RhAl_2_O_2–3_^+^ clusters with CO. Time-of-flight mass spectra for reactions of mass selected RhAl_2_O_2–3_^+^ with He (**a**), N_2_ (**c**) and CO (**b**,**d**) are shown. The time period was ∼0.6 ms for both reactions.

The RhAl_2_O_*m*_^+^ (*m*=2–6) cluster ions were generated by laser ablation of a mixed-metal disk compressed with Rh and Al powders. The generated RhAl_2_O_*m*_^+^ cluster ions were mass-selected, cooled and then interacted with N_2_ and CO in an ion trap reactor, as shown in Figs 1 and 2. On the interaction of RhAl_2_O_6_^+^ with 150 mPa N_2_ (Fig. 1a), weak N_2_ adsorption (RhAl_2_O_6_N_2_^+^) and N_2_/O_2_ exchange (RhAl_2_O_4_N_2_^+^) products were generated. Generation of RhAl_2_O_4_N_2_^+^ suggests the possible presence of superoxide (O_2_^−·^) or peroxide (O_2_^2−^) unit in RhAl_2_O_6_^+^ (RhAl_2_O_6_^+^ + N_2_ → RhAl_2_O_4_N_2_^+^ + O_2_). In sharp contrast, on the interaction of RhAl_2_O_6_^+^ with CO (Fig. 1b–d), a series of products, from RhAl_2_O_5_^+^ to RhAl_2_O^+^, were generated gradually with the increase of CO partial pressure from 2 to 13 mPa. Signals RhAl_2_O_1–5_^+^ did not appear on the interaction of RhAl_2_O_6_^+^ with even high pressure N_2_ (Fig. 1a). Additional experimental techniques such as multiphoton ionization^28^,^29^ employing pulsed lasers are required to observe the neutral CO_2_ molecules. However, N_2_ experiment in Fig. 1a also indicates that products RhAl_2_O_1–5_^+^ are due to the chemical reactions of RhAl_2_O_6_^+^ with CO rather than collision-induced dissociation and RhAl_2_O_6_^+^ may oxidize five CO molecules consecutively (equation (1)).


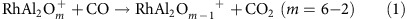


**Figure 3:**
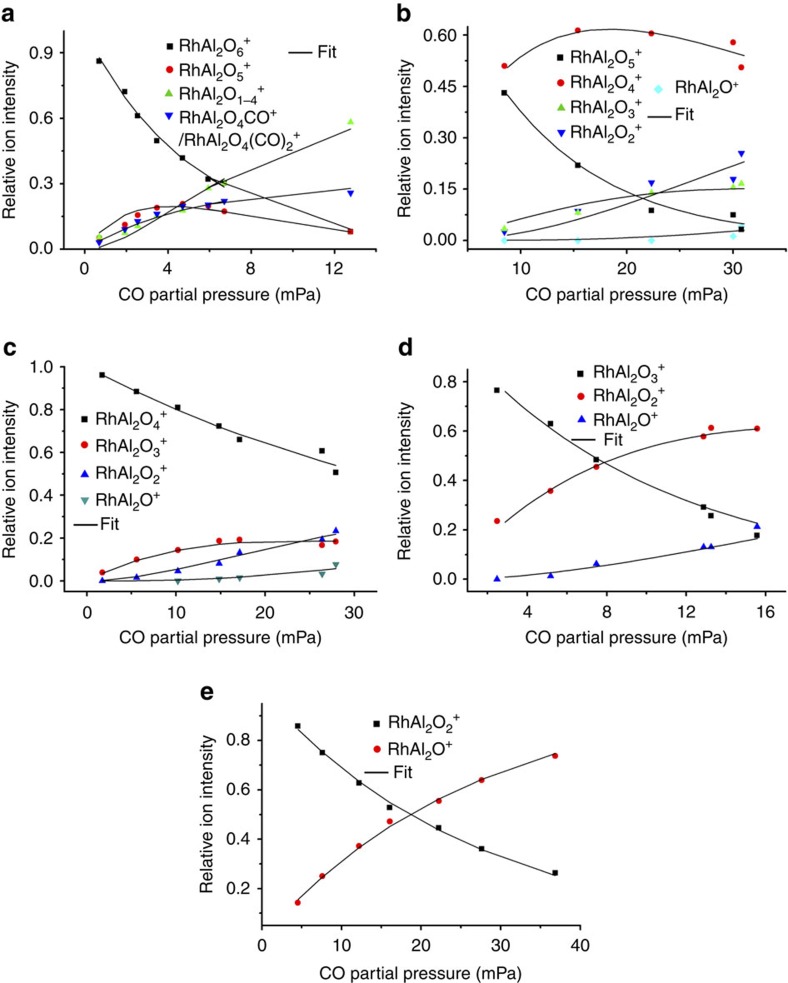
Reaction kinetics. Variation of ion intensities with respect to the partial pressures of CO in RhAl_2_O_*m*_^+^ (*m*=6−2) + CO are shown (**a**–**e**). The solid lines are fitted to the experimental data points by the least-square procedure. The Rh^+^ (1,0,0) ions (Fig. 2) are mostly generated during cooling of the RhAl_2_O_3_^+^ and RhAl_2_O_2_^+^ cluster ions through collisions with He gas in the ion trap reactor; thus, the ion intensity of Rh^+^ (nearly independent on the CO partial pressure) is excluded in the fitting. See Supplementary Table 1 for details of the determined rate constant values.

The strong signals that can be assigned as RhAl_2_O_4_CO^+^ and RhAl_2_O_4_(CO)_2_^+^ (Fig. 1a–d) on the interaction of RhAl_2_O_6_^+^ with CO indicate the displacement of the O–O unit in RhAl_2_O_6_^+^ by CO, which is more facile than N_2_ displacement (Fig. 1a), and further demonstrates the presence of O_2_^−·^ or O_2_^2−^ unit in RhAl_2_O_6_^+^. Each of the cluster source generated RhAl_2_O_*m*_^+^ (*m*=5−2) clusters could also react with CO to generate products, from RhAl_2_O_*m*−1_^+^ to RhAl_2_O^+^ (Figs 1f,h and 2b,d). This provides convincing evidence that RhAl_2_O_6_^+^ can indeed oxidize five CO molecules consecutively. The pseudo-first-order rate constants (*k*_1_, in unit of 10^−10^ cm^3^ per molecule per second) on the interaction of RhAl_2_O_*m*_^+^ (*m*=6–2) cluster ions with CO can be well fitted (Fig. 3) and the determined rate constants are presented in Supplementary Table 1. The rate constants for the reactions of the cluster source generated RhAl_2_O_*m*_^+^ (*m*=6–2) with CO are 4.9±1.5 (*m*=6), 6.2±1.9 (*m*=5), 1.6±0.5 (*m*=4), 6.9±2.0 (*m*=3) and 2.4±0.7 (*m*=2), which correspond to the reaction efficiencies^30^ of about (37±11)%, (47±14)%, (13±4)%, (54±16)% and (19±6)%, respectively. Furthermore, we note that the clusters with odd number of oxygen atoms such as RhAl_2_O_5_^+^ are more reactive towards CO oxidation than clusters with even number of oxygen atoms such as RhAl_2_O_6_^+^.

### Reaction mechanism

**Figure 4:**
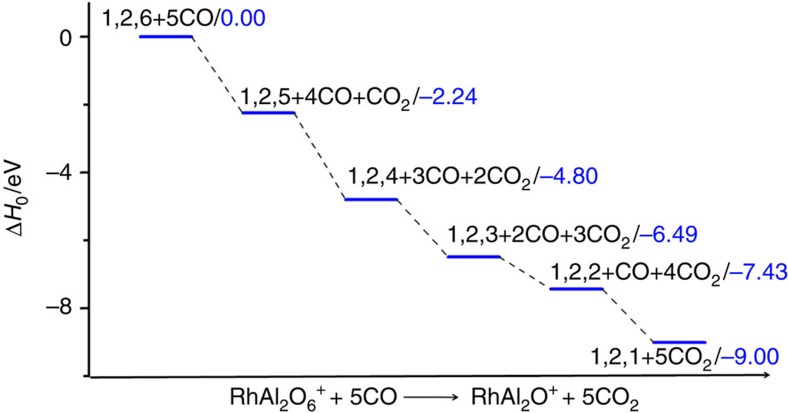
Reaction thermodynamics. Density functional theory (DFT)-calculated thermodynamic data for CO oxidation by RhAl_2_O_2–6_^+^. The energies are zero-point vibration-corrected in unit of eV.

**Figure 5:**
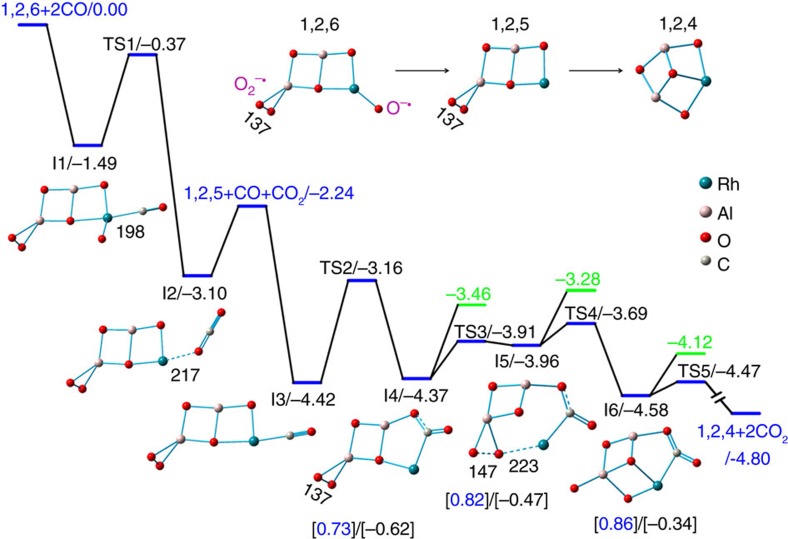
Structures and reaction mechanisms. Density functional theory (DFT)-calculated potential energy profiles for the oxidation of the first two CO molecules by RhAl_2_O_6_^+^. The lowest energy structure of RhAl_2_O_6_^+^ (1,2,6) and the products ^*P*^RhAl_2_O_5_^+^ (1,2,5) and RhAl_2_O_4_^+^ (1,2,4) are provided. Symbols O_2_^−·^ and O^−·^ denote superoxide and atomic oxygen radical species, respectively. The relative energies for intermediates (I1–I6) and transition states (TS1–TS5) are in unit of eV. Structures of I1–I6 are shown. Bond lengths are given in pm. The values in green show the relative energies for direct CO_2_ desorption from I4 to I6. The values in the square brackets show the natural charges on Rh (blue) and CO_2_ unit (black). See also Supplementary Figs 9–13 for more information.

The density functional theory calculated thermodynamic data for CO oxidation by RhAl_2_O_*m*_^+^ (*m*=2–6) are shown in Fig. 4. The overall oxidation (RhAl_2_O_6_^+^ + 5CO → RhAl_2_O^+^ + 5CO_2_) is highly exothermic (−9.00 eV). The low-lying energy isomers of clusters RhAl_2_O_*m*_^+^ (*m*=6–1) are provided in Supplementary Figs 1–6. The lowest energy isomer of RhAl_2_O_6_^+^ is in the triplet spin state (Supplementary Fig. 1) and contains a superoxide O_2_^−·^ unit (O–O bond: 137 pm; Fig. 5). The existence of O_2_^−·^ unit in RhAl_2_O_6_^+^ is consistent with the appearance of RhAl_2_O_4_N_2_^+^ and RhAl_2_O_4_CO^+^ (or RhAl_2_O_4_(CO)_2_^+^) on the interaction of RhAl_2_O_6_^+^ with N_2_ and CO (Fig. 1a–d), respectively. Supplementary Figs 7 and 8 show that the Al-site adsorption contributes to the displacement of the O_2_^−·^ unit in RhAl_2_O_6_^+^ by N_2_ or CO and both reactions are calculated to be thermodynamically and kinetically favourable, and CO displacement is more facile than N_2_ displacement. This is consistent with the relatively higher intensity of RhAl_2_O_4_CO^+^ (or RhAl_2_O_4_(CO)_2_^+^) than RhAl_2_O_4_N_2_^+^ observed in the experiment (Fig. 1a–d). The positively charged Rh in RhAl_2_O_6_^+^ (natural charge: +1.14 *e*) can trap CO tightly at the first step (I1, Δ*H*_0_=−1.49 eV; Fig. 5) and then the oxidation of CO (I1→TS1→I2) by the highly reactive atomic oxygen radical anion O^−·^ (ref. 31) takes place. Direct CO oxidation by the O_2_^−·^ unit has to suffer from a positive barrier of 0.03 eV, which is much less favourable than the oxidation by O^−·^. The Rh atom in product RhAl_2_O_5_^+^ (denoted as ^*P*^RhAl_2_O_5_^+^, the structure of which is different from the lowest energy structure; Supplementary Fig. 2) can capture another CO tightly (I3, binding energy of −2.18 eV). Formation of the bent CO_2_ is the bottleneck (I3→TS2→I4) for CO oxidation by ^*P*^RhAl_2_O_5_^+^. This step is to subject to a barrier of 1.26 eV. The subsequent steps follow a nearly downhill pathway characterized by small barriers to yield RhAl_2_O_4_^+^ and CO_2_ (Supplementary Fig. 9). Furthermore, theoretical calculations show that the energy of the critical transition state for reaction RhAl_2_O_5_^+^ + CO (−0.47 eV; Supplementary Fig. 10) is lower with respect to that for reaction RhAl_2_O_6_^+^ + CO (TS1, −0.37 eV; Fig. 5). This can well interpret the more reactive behaviour of the cluster source-generated RhAl_2_O_5_^+^ than RhAl_2_O_6_^+^ in the experiment.

The key step for the transfer of five oxygen atoms from RhAl_2_O_6_^+^ to CO lies in the facile dissociation of the O_2_^−·^ unit in ^*P*^RhAl_2_O_5_^+^. Dissociation of the chemically adsorbed molecular O_2_ (superoxide O_2_^−·^ or peroxide O_2_^ 2−^) is often considered to be the crucial step in oxidation reactions^32^. Recent gas-phase studies indicated that a single Au atom in AuTi_3_O_8_^−22^ is not enough to promote the dissociation of the O_2_^ 2−^ unit, while the Au dimer in Au_2_VO_4_^−^ can promote O_2_^ 2−^ unit dissociation or direct participation in CO oxidation^25^. In this study, a single Rh atom in ^*P*^RhAl_2_O_5_^+^ can promote the dissociation of the O_2_^−·^ unit and the process is much more favourable than CO_2_ desorption (Fig. 5). This is rationalized by the strong Rh–C multiple bonds (5.97 eV)^33^ and the strong Rh–O bond (4.16 eV)^34^. Thus, the Al–O_2_^−·^ unit in I4 can approach the Rh atom favourably to form structure Al–O_2_^ 2−^˙̇̇Rh–CO_2_ in I5. The elongation of the O–O bond from 137 pm in I4 to 147 pm in I5 is a good indicator for the activation of the superoxide O_2_^−·^ to peroxide O_2_^ 2−^ unit. The structure of I5 is crucial to induce further electron flowing into the O_2_^ 2−^ unit from both of the Rh atom and the CO_2_ unit (Fig. 5), and then the O_2_^ 2−^ unit can be dissociated favourably to produce O^2−^–Al–O^2−^–Rh–CO_2_ (I5→TS4→I6). Direct oxidation of CO by the O_2_^−·^ unit in ^*P*^RhAl_2_O_5_^+^ (Supplementary Fig. 9) is less favourable than the pathway in Fig. 5. This is consistent with previous study that instead of the direct participation in CO oxidation, molecular oxygen adsorbs at the interface between the oxygen vacancy and the single Rh site and then is followed by facile dissociation^35^. Release of three additional oxygen atoms from the resulting RhAl_2_O_4_^+^ to CO are calculated to be thermodynamically and kinetically favourable (Supplementary Figs 11–13). In each of these OAT steps, Rh atom functions as the preferred trapping site to anchor CO and then delivers CO for oxidation by the oxygen atoms in direct connection with Rh. The theoretical calculations well interpret the unique reactivity of RhAl_2_O_6_^+^ observed in the experiment.

## Discussion

Metal-mediated OAT reaction is usually accompanied with the reduction of central metal by electrons that are stored originally in the removed oxygen atoms^36^ (equation (2)).


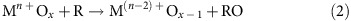


**Figure 6:**
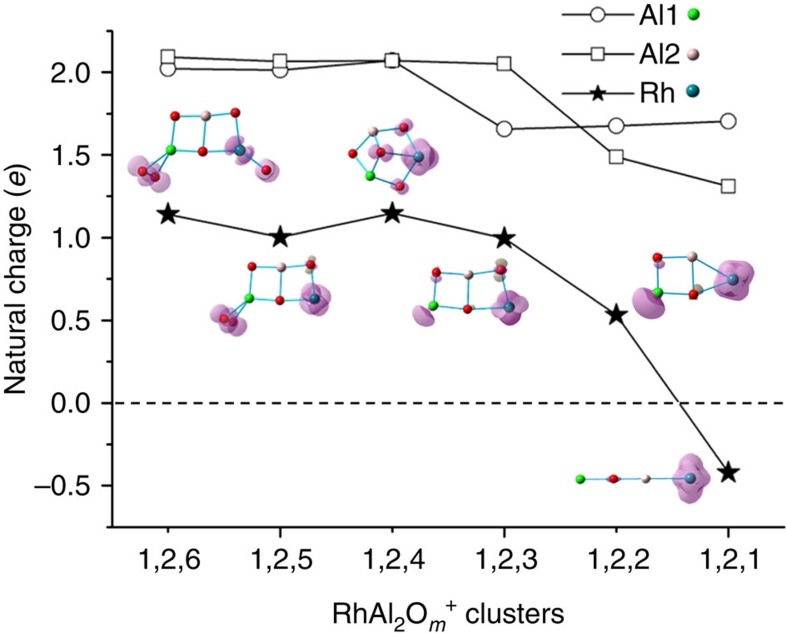
Natural charge. Density functional theory (DFT)-calculated natural charges (*e*) on the Rh atom and Al atoms in RhAl_2_O_1–6_^+^. The spin density distributions on individual atoms are shown in the purple isosurfaces.

The positively charged metal centre is crucial to provide not only the characteristic site for the adsorption of CO (ref. 37) but also the oxidative centre to accept electrons. Recent gas-phase studies highlighted that the cleavage of Au–O bond and the formation of Au–*M* bond (*M*=Al, V, Ti, and Fe) is of great importance in CO oxidation by Au-doped clusters^22^,^23^,^24^,^25^. However, each of the Au-doped clusters can oxidize only one or at most two CO molecules and then the polarity conversion of Au atom from positive to negative takes place because of the formation of the reductive Au–*M* bond. In sharp contrast, natural charge analysis demonstrates that after the transfer of four oxygen atoms from RhAl_2_O_6_^+^ to CO, the Rh atom is still positively charged (+0.53*e*, Fig. 6) in product RhAl_2_O_2_^+^, which can also oxidize a CO molecule. The natural charge on Rh atom is decreased from +1.14*e* in RhAl_2_O_6_^+^ to +1.00*e* in ^*P*^RhAl_2_O_5_^+^ after the oxidation of the first CO. In this step, Rh acts as the primary centre to accumulate the electron that is localized originally on O^−·^ radical, as shown from the change of spin density distribution, RhAl_2_O_6_^+^ versus ^*P*^RhAl_2_O_5_^+^. However, the Rh atom is re-oxidized in product RhAl_2_O_4_^+^ (+1.15*e*) after the oxidation of the second CO due to the dissociation of the O_2_^−·^ unit (I4→I5→I6; Fig. 5). This step is crucial to recover the oxidative reactivity of Rh. *In situ* Raman spectroscopic study also demonstrated that supported Rh oxide can oxidize CO and then the Rh oxide is subsequently re-oxidized by the oxygen atoms from oxide support^7^. This phenomenon can be traced back to the well-fitting strength of Rh–O bond (4.16 eV)^34^, which is strong enough to promote the dissociation of the O_2_^−·^ unit in^*P*^RhAl_2_O_5_^+^ and prevent the formation of the reductive Rh–Al bond (3.26 eV, by theoretical calculation) in RhAl_2_O_2–6_^+^ but at the same time is relatively weak to deliver oxygen atoms to oxidize CO (O–CO: 5.52 eV)^38^. Previous studies show that Rh prefers to coordinate not only with the surface oxygen atoms but also the subsurface oxygen in oxide support^35^. The strong Rh–O bond inhibits the migration of Rh atom to the oxygen vacancy and remains positively charged. Using high-resolution *in situ* X-ray diffraction and transmission electron microscopy, Stierle and colleagues^1^ reported the reversible and oxygen-induced shape transformation of Rh nanoparticles by the formation of O–Rh–O surface oxide during the cycle of catalytic CO oxidation. In contrast, the stronger Au–M (Au–Al=3.37 eV (ref. 39), Au–V=2.49 eV (ref. 38) and Au–Ti=2.56 eV (by theoretical calculation)) than Au–O bond (2.27 eV)^34^ facilitates the formation of the reductive Au–M bond after the oxidation of only one or two CO molecules.

The negatively charged Rh has been theoretically predicted^40^ and experimentally postulated^41^. The RhAl_2_O^+^ cluster is a linear structure (Al–O–Al–Rh) and the single oxygen atom is sandwiched between two Al atoms. This structure with triplet spin state has been confirmed to be the lowest energy isomer of RhAl_2_O^+^ by more accurate CCSD(T) calculation (Supplementary Fig. 6). The two unpaired electrons are mainly localized on the Rh atom (∼1.83 *μ*_B_, Fig. 6), indicating that such Rh atom can be considered to be Rh^−1^ (4*d*^9^5*s*^1^). The electron configuration calculations (4*d*^8.75^5*s*^0.64^5*p*^0.03^) provide solid evidence for the negatively charged Rh in RhAl_2_O^+^. Thus, the oxidation state of Rh changes from +2.5 in RhAl_2_O_6_^+^ to −1 in RhAl_2_O^+^ (calculated based on the distribution of spin density; Fig. 6) during the transfer of five oxygen atoms from RhAl_2_O_6_^+^ to CO. This rather large range of Rh oxidation state changes in chemical reactions has rarely been reported (the Au oxidation state changes from +1 to −1), covering from cationic to anionic, and it is the driving force to accumulate the electrons that are stored originally in the released oxygen atoms and promotes the unique oxidative reactions to proceed.

In conclusion, we have demonstrated that a single atom of Rh can unexpectedly promote the transfer of five oxygen atoms to oxidize CO from a nine-atom cluster RhAl_2_O_6_^+^. This study leads a leap ahead towards OAT reactions in the field of cluster science and represents an important step to understand the participation of lattice oxygen promoted by noble metals. The preferable Rh–O rather than the reductive Rh–Al bond formation together with the capability of Rh to accumulate the large amounts of electrons are crucial factors to drive the unique reactions. This gas-phase study reveals the molecular-level origin for the puzzling experimental observation that trace amounts of Rh can promote the reactivity of lattice oxygen of Al_2_O_3_ (refs 4, 5, 9), a chemically very inert material.

## Methods

### Cluster generation and reactivity detection

The Rh_*x*_Al_*y*_O_*z*_^+^ cluster ions were generated by laser ablation of a mixed-metal disk compressed with Rh and Al powders (molar radio Rh/Al=1/1) in the presence of O_2_ (0.4%) seeded in a He carrier gas with a backing pressure of 6.0 standard atmospheres. The cluster ions of interest were mass selected using a quadrupole mass filter and then entered into a linear ion trap (LIT) reactor, where they were cooled by collisions with a pulse of He gas and then interacted with a pulse of 5% (for reactions RhAl_2_O_*m*_^+^ (*m*=2–5) + CO) or 2% (for reaction RhAl_2_O_6_^+^ + CO) CO seeded in He for around 0.6∼1.1 ms. The temperature of cooling gas (He), reactant gases (CO or N_2_) and the LIT reactor was around 298 K. The cluster ions ejected from the LIT were detected by a reflector time-of-flight mass spectrometer. The details of running the time-of-flight mass spectrometer^42^, quadrupole mass filter^43^ and the LIT^44^ can be found in our previous works.

### Rate constant fitting

Equation (3) was used to determine the pseudo-first-order rate constants (*k*_1_) of cluster reactions in an ion trap reactor^44^, in which *I*_R_ is the signal intensity of the reactant cluster ions, *I*_T_ is the total ion intensity including product ion contribution, *k*_B_ is the Boltzmann constant, *T* is the temperature (∼298 K), *t*_R_ is the reaction time and *P* is the effective pressure of the reactant gas in the ion trap reactor.


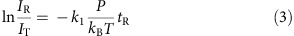


To calculate the reaction efficiencies (the possibilities of reaction on each collision), the collision rate constants were calculated on the basis of the surface charge capture model developed in the literature^30^. It is noteworthy that for reaction RhAl_2_O_5_^+^ + CO, the relative ion intensity of RhAl_2_O_5_^+^ is the reactive component generated in the experiment and the unreactive component is not included. The unreactive component of RhAl_2_O_5_^+^ could be well-fitted by equation (4)^45^.


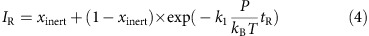


in which *x*_inert_ is the relative intensity of the unreactive component of RhAl_2_O_5_^+^ and *k*_1_ is the pseudo-first-order rate constant of the reactive component of RhAl_2_O_5_^+^. The *x*_inert_ was determined to be about 12%, indicating that the experimentally generated RhAl_2_O_5_^+^ may have isomers that are not or less reactive with CO.

### Computational details

Density functional theory calculations using the Gaussian 09 (ref. 46) programme were carried out to investigate the mechanistic details on the oxidation of five CO molecules by a nine-atom rhodium–aluminum oxide cluster (RhAl_2_O_6_^+^). To find an appropriate functional for the Rh–Al–O system, the bond dissociation energies of Rh–O, Rh–C, Al–O, O–O, Rh–Al and O–CO were computed by various functionals and compared with available experimental data (Supplementary Table 2). It turns out that M06L^47^ was the best overall; thus, the results by M06L were given throughout the work. The TZVP basis set^48^ for Al, C and O atoms and a D95V basis set^49^ combined with the Stuttgart/Dresden relativistic effective core potential (denoted as SDD in Gaussian software) for Rh atom were used in all the calculations. A Fortran code based on a genetic algorithm^50^ was used to generate initial guess structures of RhAl_2_O_1–6_^+^. The reaction mechanisms were studied for RhAl_2_O_6_^+^ + 5CO → RhAl_2_O^+^ + 5CO_2_. The relaxed potential energy surface scan was used extensively to obtain good guess structures for intermediates and transition states along the pathways. The transition states were optimized using the Berny algorithm^51^. Intrinsic reaction coordinate calculations^52^,^53^ were performed so that each transition state connects two appropriate local minima. Vibrational frequency calculations were carried out to check that intermediates and transition state have zero and only one imaginary frequency, respectively.

## Additional information

**How to cite this article:** Li, X.-N. *et al*. A nine-atom rhodium–aluminum oxide cluster oxidizes five carbon monoxide molecules. *Nat. Commun.* 7:11404 doi: 10.1038/ncomms11404 (2016).

## Supplementary Material

Supplementary Information Supplementary Figures 1-13 and Supplementary Tables 1-2
