# Supplementary material for: A nine-atom rhodium–aluminum oxide cluster oxidizes five carbon monoxide molecules
Source: Nat Commun. 2016 Apr 20;7:11404. doi: 10.1038/ncomms11404 (PMC4843021; doi:10.1038/ncomms11404)
Supplement: Supplementary Information — Supplementary Figures 1-13 and Supplementary Tables 1-2 [file ncomms11404-s1.pdf]

Supplementary Figures

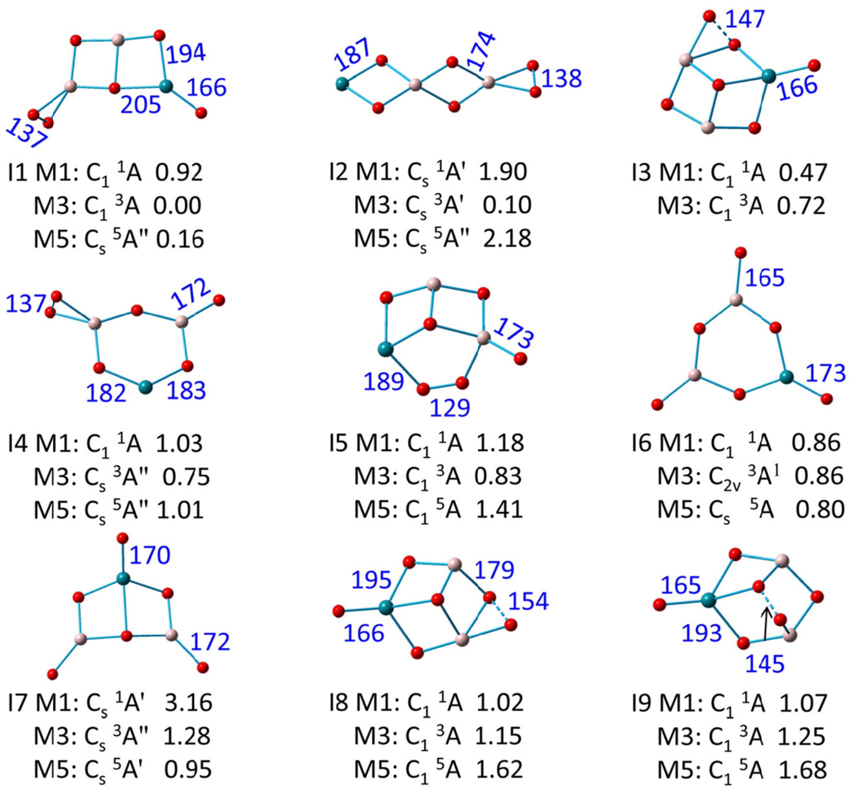

Supplementary Figure 1. DFT calculated structures and relative energies of the $RhAl_2O_6^+$ isomers. The energies are in unit of eV and bond lengths are given in pm.

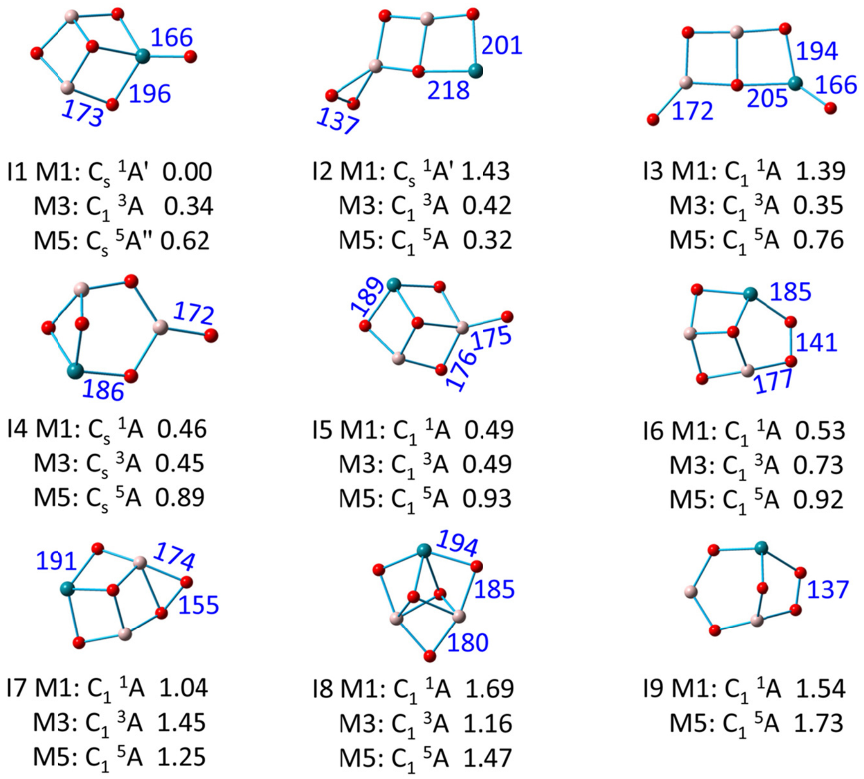

Supplementary Figure 2. DFT calculated structures and relative energies of the $RhAl_2O_5^+$ isomers. The energies are in unit of eV and bond lengths are given in pm.

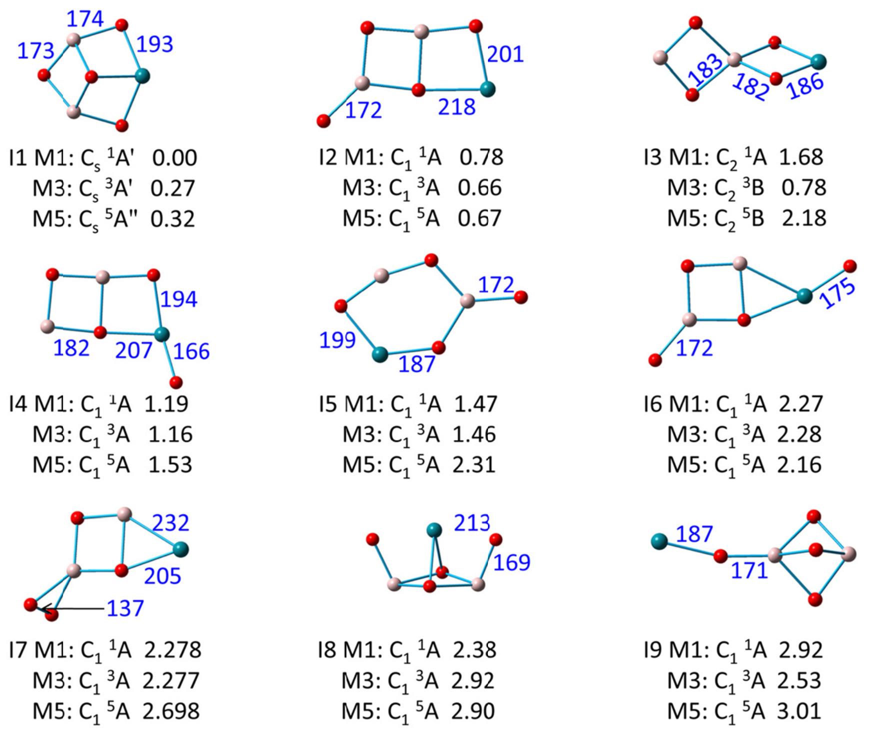

Supplementary Figure 3. DFT calculated structures and relative energies of the $RhAl_2O_4^+$ isomers. The energies are in unit of eV and bond lengths are given in pm.

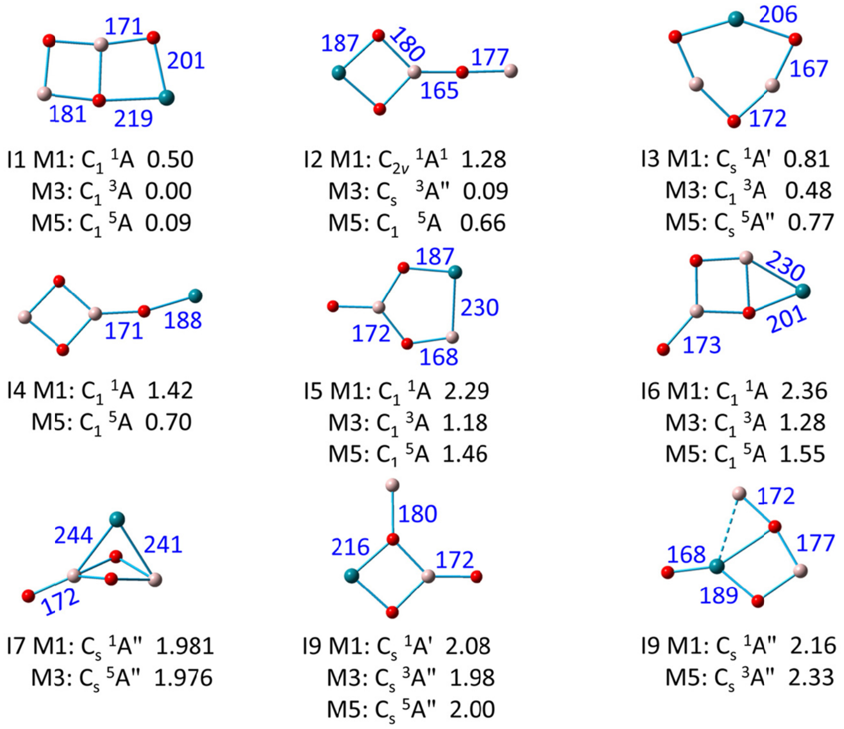

Supplementary Figure 4. DFT calculated structures and relative energies of the $RhAl_2O_3^+$ isomers. The energies are in unit of eV and bond lengths are given in pm.

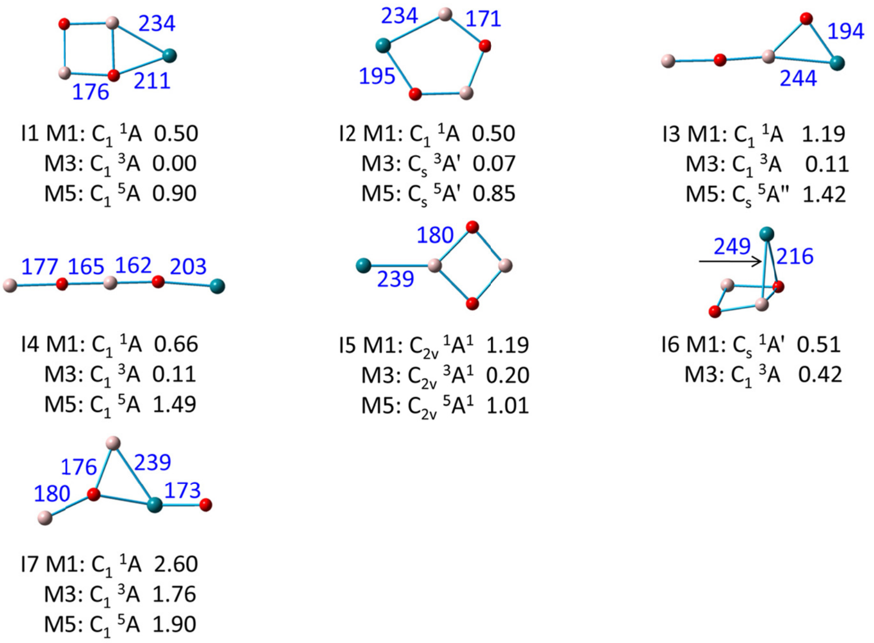

Supplementary Figure 5. DFT calculated structures and relative energies of the $RhAl_2O_2^+$ isomers. The energies are in unit of eV and bond lengths are given in pm.

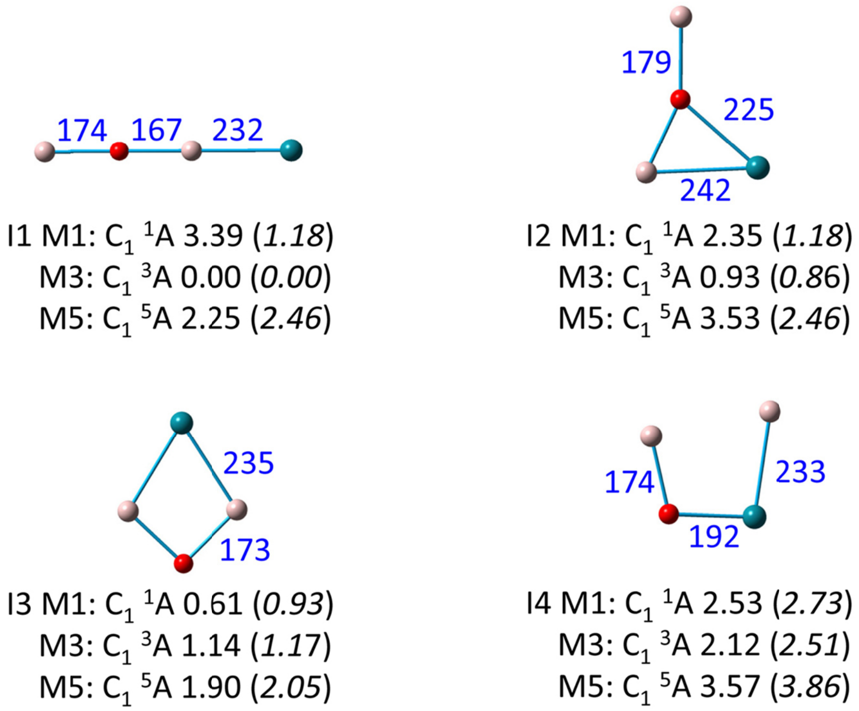

Supplementary Figure 6. DFT calculated structures and relative energies of the $RhAl_2O^+$ isomers. The energies are in unit of eV and bond lengths are given in pm. Single point energies calculated at the CCSD (T) level are listed in the parentheses.

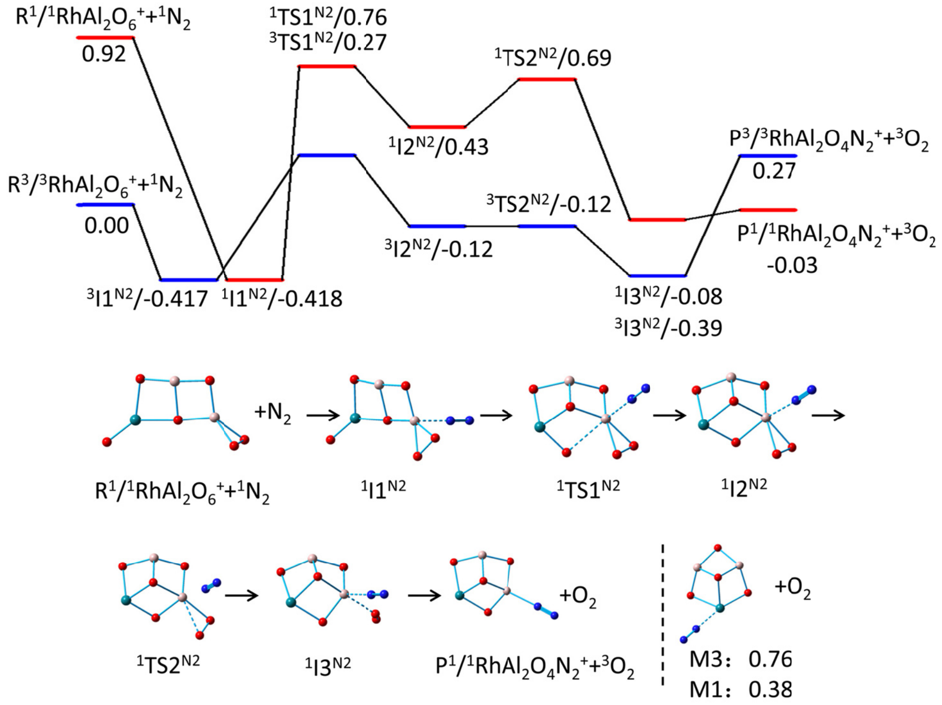

Supplementary Figure 7. DFT calculated potential energy profile for reaction $RhAl_2O_6^+ + N_2 \rightarrow RhAl_2O_4N_2^+ + O_2$. The relative energies are in unit of eV. Bond lengths are given in pm.

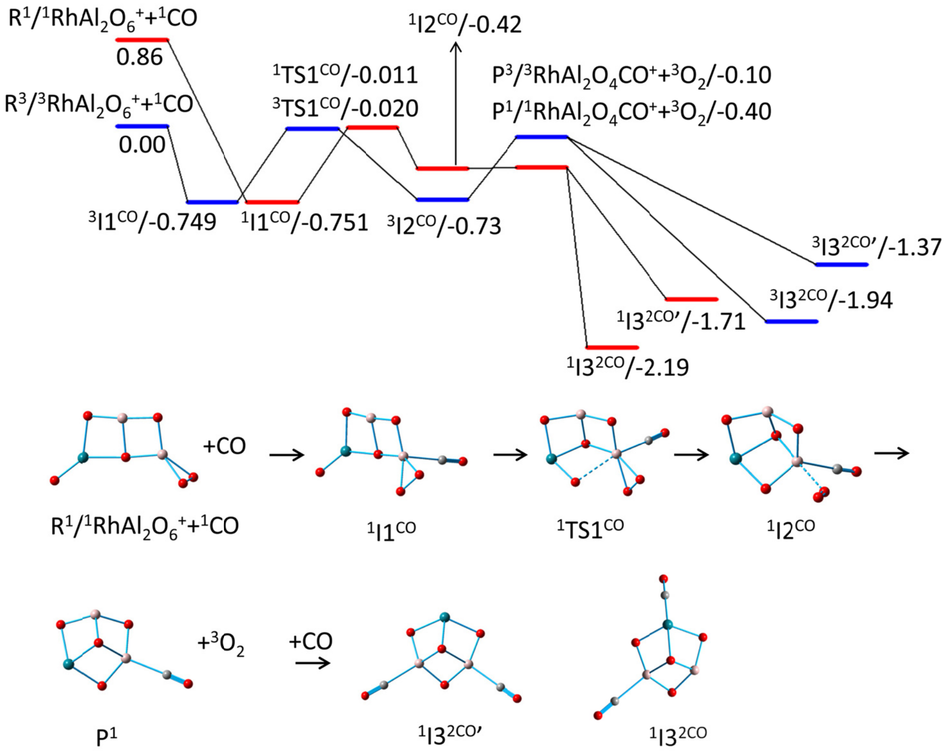

Supplementary Figure 8. DFT calculated potential energy profile for reaction $RhAl_2O_6^+ + 2CO \rightarrow RhAl_2O_4(CO)_2^+ + O_2$. The relative energies are in unit of eV. Bond lengths are given in pm.

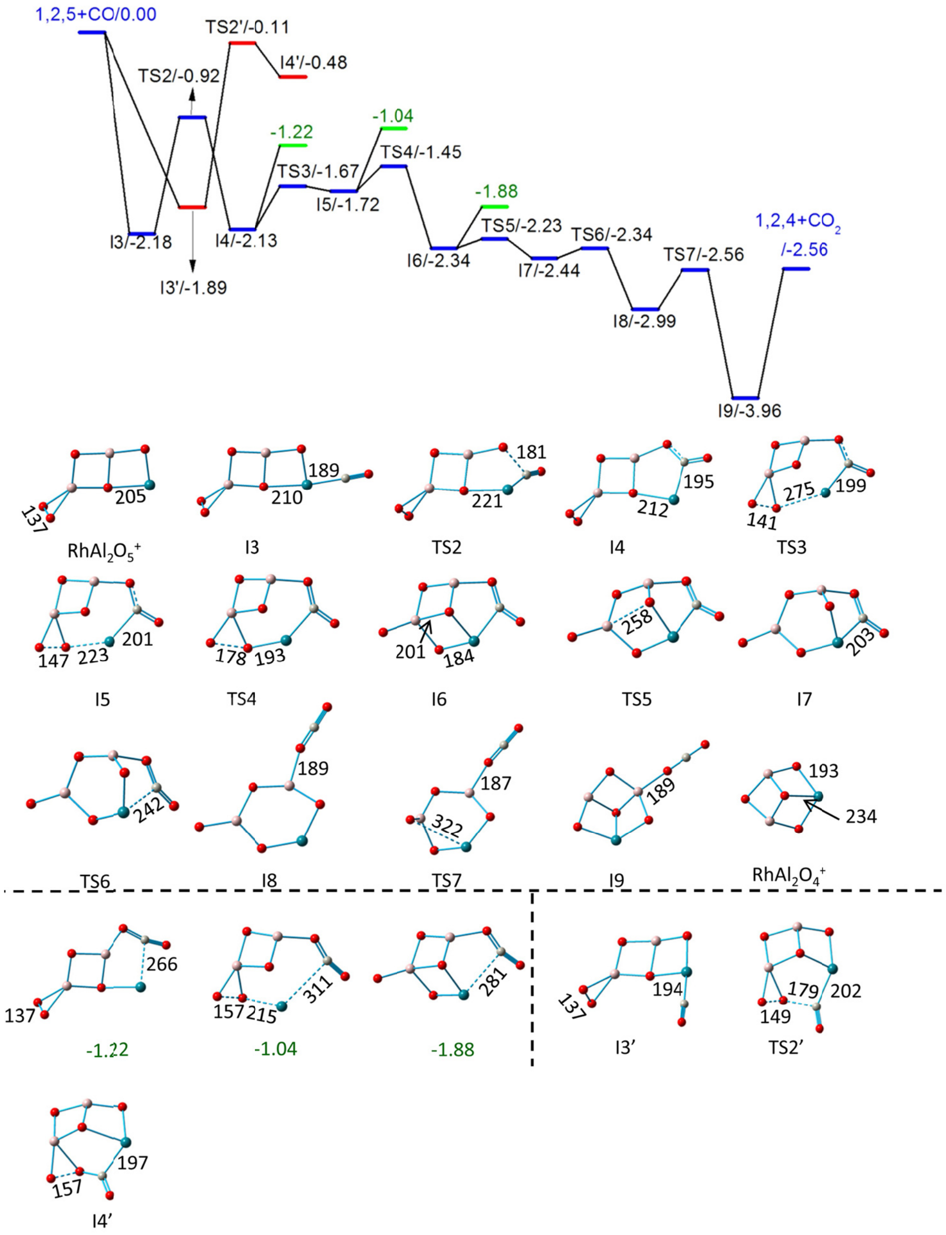

Supplementary Figure 9. DFT calculated potential energy profile for reaction $^{P}RhAl_2O_5^+ + CO \rightarrow RhAl_2O_4^+ + CO_2$ on the triplet state. The relative energies are in unit of eV. Bond lengths are given in pm.

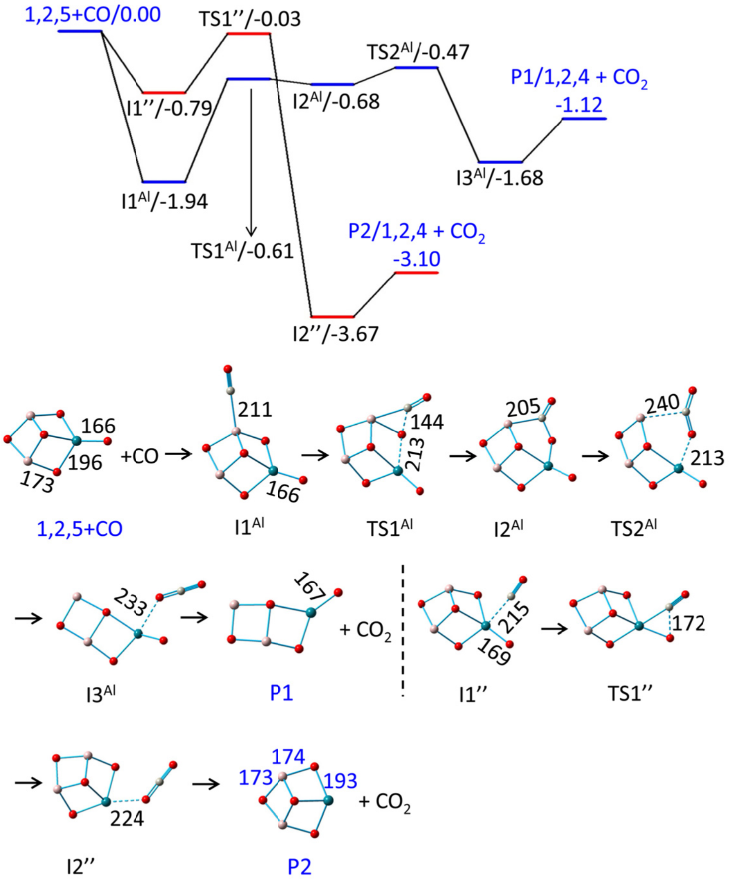

Supplementary Figure 10. DFT calculated potential energy profile for $RhAl_2O_5^+$ + CO → $RhAl_2O_4^+$ + CO_2 on the singlet state. The relative energies are in unit of eV. Bond lengths are given in pm.

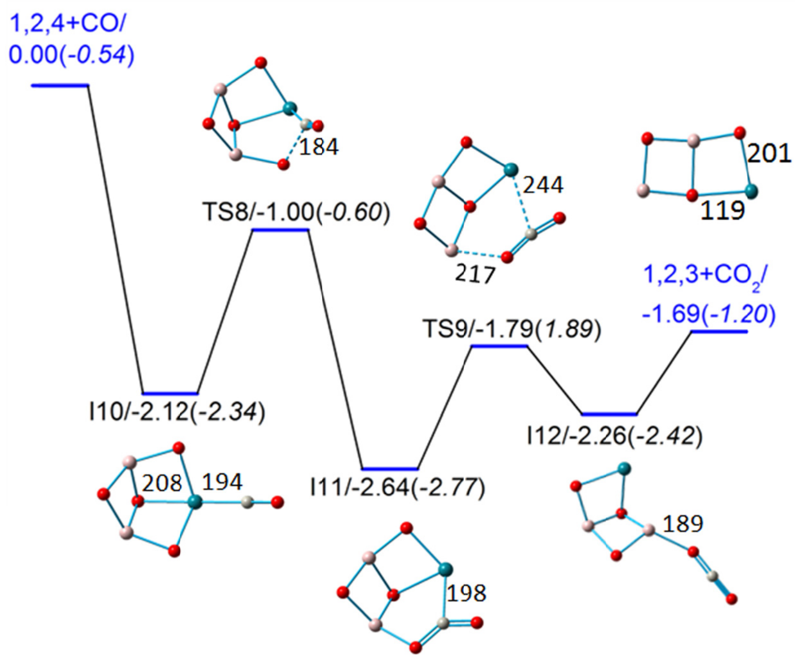

Supplementary Figure 11. DFT calculated potential energy profile for reaction $RhAl_2O_4^+$ + CO → $RhAl_2O_3^+$ + CO_2 on the triplet state. The corresponding singlet state energies are listed in the parentheses. The relative energies are in unit of eV. Bond lengths are given in pm.

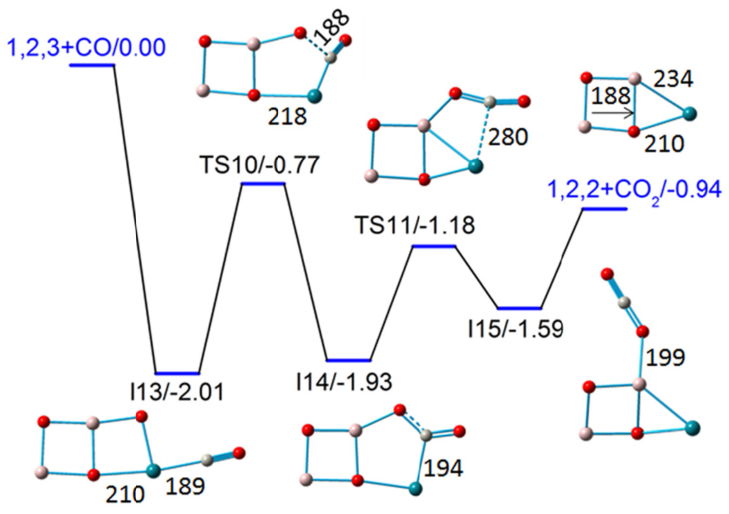

Supplementary Figure 12. DFT calculated potential energy profile for reaction $RhAl_2O_3^+ + CO \rightarrow RhAl_2O_2^+ + CO_2$ on the triplet state. The relative energies are in unit of eV. Bond lengths are given in pm.

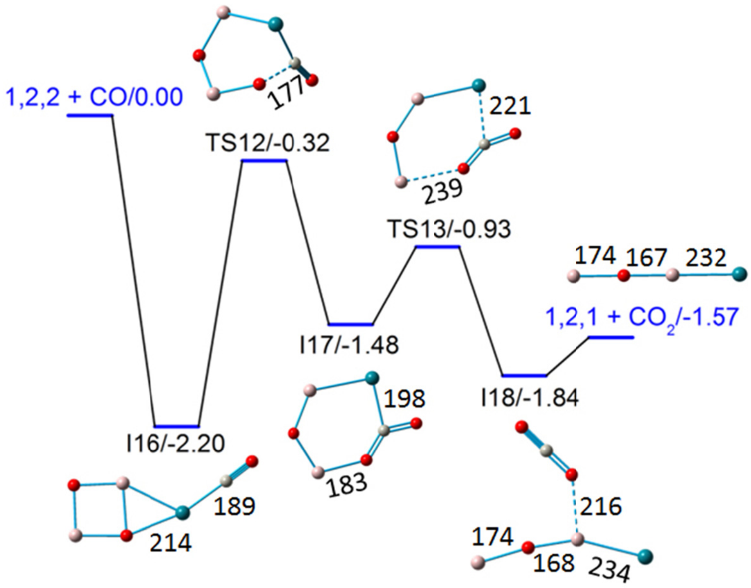

Supplementary Figure 13. DFT calculated potential energy profile for reaction $RhAl_2O_2^+$ + CO → $RhAl_2O^+$ + CO_2 on the triplet state. The relative energies are in unit of eV. Bond lengths are given in pm.

Supplementary Tables

Supplementary Table 1. Pseudo-first-order rate constants (k_1, 10^{-10} cm^3 $molecule^{-1}s^{-1}$) determined from reactions of cluster source generated $RhAl_2O_m^+$ (m = 6-2) with CO. The uncertainties of the absolute and relative rate constants are within ±40% and ±20%, respectively.

Mass-selected clusters	$RhAl_2O_6^+$	$RhAl_2O_5^+$	$RhAl_2O_4^+$	$RhAl_2O_3^+$	$RhAl_2O_2^+$
$RhAl_2O_6^+$+CO	4.9±1.5				
$RhAl_2O_5^+$+CO	11.3±3.4	6.2±1.9			
$RhAl_2O_4^+$+CO		1.6±0.5	1.6±0.5		
$RhAl_2O_3^+$+CO		5.7±1.7	5.0±1.5	6.9±2.0	
$RhAl_2O_2^+$+CO		0.7±0.2	1.6±0.5	1.9±0.6	2.4±0.7
$RhAl_2O_6^+$+CO→ $RhAl_2O_4CO^+$+O_2	2.2±0.66				

Supplementary Table 2. Bond energies by experiments and different functionals. The values are in unit of eV.

		Rh-O	Rh-C	Al-O	O-O	Rh-Al	O-CO
Experiments	Values	4.16	5.97	5.27	5.13		5.52
Hybrid Functionals	B3LYP	4.062	5.563	4.921	5.111	3.036	5.443
	B1B95	3.963	5.594	4.854	5.151	3.115	5.522
	B3P86	4.248	5.89	5.052	5.379	3.248	5.745
	B1LYP	3.727	5.193	4.713	4.827	2.856	5.229
	M06	4.242	5.910	4.869	4.952	3.307	5.697
	M062X	3.339	5.089	4.908	4.999	2.947	5.317
	X3LYP	4.012	5.504	4.901	5.089	3.017	5.437
	PBE1PBE	3.901	5.558	4.761	5.127	3.056	5.590
	B3PW91	4.047	5.684	4.851	5.168	3.113	5.576
	O3LYP	4.071	5.683	4.891	5.356	2.872	5.607
	wB97	2.921	5.926	4.804	5.214	3.59	5.387
Pure Functionals	**M06L**	**4.235**	**5.941**	**5.041**	**5.170**	**3.256**	**5.774**
	TPSS	4.289	5.917	5.121	5.284	3.113	5.576
	BLYP	4.926	6.437	5.352	5.680	3.418	5.723
	BP86	5.091	5.340	5.439	5.939	3.624	6.014
	BPW91	4.929	6.605	5.300	5.768	3.529	5.917
	BPBE	4.944	6.630	5.296	5.791	3.540	5.935
